# Toward a Platform for the Treatment of Burns: An Assessment of Nanoemulsions vs. Nanostructured Lipid Carriers Loaded with Curcumin

**DOI:** 10.3390/biomedicines11123348

**Published:** 2023-12-18

**Authors:** Gabriela de Moraes Soares Araújo, Ana Isabel Sá Loureiro, Jamile Lima Rodrigues, Paula Alice Bezerra Barros, Priscila Cristina Bartolomeu Halicki, Daniela Fernandes Ramos, Marcelo Augusto Germani Marinho, Daniela Pastorim Vaiss, Gustavo Richter Vaz, Virginia Campello Yurgel, Juliana Bidone, Ana Luiza Muccillo-Baisch, Mariana Appel Hort, Artur Manuel Cavaco Paulo, Cristiana Lima Dora

**Affiliations:** 1 Graduate Program in Health Sciences, Federal University of Rio Grande, Rio Grande 96203-900, RS, Brazil; gabrieladmsaraujo@gmail.com (G.d.M.S.A.); alicebarros.pb@gmail.com (P.A.B.B.); priscilahalicki@hotmail.com (P.C.B.H.); daniferamos@gmail.com (D.F.R.); danipvaiss@gmail.com (D.P.V.); richtervaz@gmail.com (G.R.V.); virginia.yurgel@gmail.com (V.C.Y.); anabaisch@gmail.com (A.L.M.-B.); marianaappel@gmail.com (M.A.H.); 2 CEB—Center of Biological Engineering, University of Minho, Campus de Gualtar, 4710-057 Braga, Portugal; aloureiro@ceb.uminho.pt; 3 Graduate Program in Food Science and Engineering, Federal University of Rio Grande, Rio Grande 96203-900, RS, Brazil; jamilelr@gmail.com; 4 Graduate Program in Physiology, Federal University of Rio Grande, Rio Grande 96203-900, RS, Brazil; marceloaugustomarinho@gmail.com; 5 Center of Chemical, Pharmaceutical, and Food Sciences, Federal University of Pelotas, Pelotas 96010-610, RS, Brazil; julianabidone@gmail.com

**Keywords:** burn, nanotechnology, curcumin, nanoemulsion, nanostructured lipid carrier

## Abstract

Curcumin is a highly promising substance for treating burns, owing to its anti-inflammatory, antioxidant, antimicrobial, and wound-healing properties. However, its therapeutic use is restricted due to its hydrophobic nature and low bioavailability. This study was conducted to address these limitations; it developed and tested two types of lipid nanocarriers, namely nanoemulsions (NE-CUR) and nanostructured lipid carriers (NLC-CUR) loaded with curcumin, and aimed to identify the most suitable nanocarrier for skin burn treatment. The study evaluated various parameters, including physicochemical characteristics, stability, encapsulation efficiency, release, skin permeation, retention, cell viability, and antimicrobial activity. The results showed that both nanocarriers showed adequate size (~200 nm), polydispersity index (~0.25), and zeta potential (~>−20 mV). They also showed good encapsulation efficiency (>90%) and remained stable for 120 days at different temperatures. In the release test, NE-CUR and NCL-CUR released 57.14% and 51.64% of curcumin, respectively, in 72 h. NE-CUR demonstrated better cutaneous permeation/retention in intact or scalded skin epidermis and dermis than NLC-CUR. The cell viability test showed no toxicity after treatment with NE-CUR and NLC-CUR up to 125 μg/mL. Regarding microbial activity assays, free curcumin has activity against *P. aeruginosa*, reducing bacterial growth by 75% in 3 h. NE-CUR inhibited bacterial growth by 65% after 24 h, and the association with gentamicin had favorable results, while NLC-CUR showed a lower inhibition. The results demonstrated that NE-CUR is probably the most promising nanocarrier for treating burns.

## 1. Introduction

Burns are injuries caused by thermal, chemical, electrical, or radioactive agents [1,2]. Burns reach the skin, partially or completely destroying its attachments. Depending on the depth, burns are characterized as first, second, or third-degree, measured by the percentage of affected body surface [2,3]. Such injuries are considered highly aggressive, physically and psychologically [4,5,6], since the pain generated in patients is caused by damage to peripheral sensory neurons and by inflammation at the site, which exacerbates an acute response [7].

A product with several pharmacological properties that allows an adequate action of the medications through the topical route is essential to treat skin burns [8,9,10]. To assist in the process of skin regeneration, formulations for burn treatments must also contain actives with anti-inflammatory and antioxidant activity. Also, since bacterial infections in wounds and burns are very common, becoming a risk to the regeneration process [11,12], it is important to use products that can help antimicrobial treatment. Curcumin has been investigated due to its pharmacological activities [13,14,15,16,17].

Curcumin is a polyphenolic compound with antioxidant and anti-inflammatory effects that have been researched for various purposes, including tissue regeneration [18,19,20,21,22,23]. Many studies have been conducted on the use of curcumin for the treatment of burns. Studies showed that curcumin can control burn pain due to its analgesic effect, in addition to improving healing mediated with anti-inflammatory mechanisms [13,24,25,26,27,28]. Studies using rats with burns showed that curcumin was able to completely re-epithelialize wounds, with a decrease in inflammatory cells, an increased proliferation of fibroblasts, and angiogenesis, showing that curcumin played a prominent role in the post-burn wound healing process [29,30]. However, curcumin is unstable in light and has low aqueous solubility, which makes it hard to incorporate into pharmaceutical forms. In this sense, nanotechnology emerges as an alternative for improving the biopharmaceutical properties of drugs, increasing solubility and stability when compared to the molecular form, in addition to improving skin permeation and solving difficulties inherent in the administration of this compound [23,31,32,33].

Nanotechnology involves the study of substances encapsulated in carriers on the nanometer scale and can be composed of different materials, such as natural or synthetic polymers, lipids, phospholipids, or metals [31,34,35,36]. The development of nanoscale biomaterials is promising for the promotion of tissue regeneration processes since the use of nanocarriers assists in the penetration of drugs due to their physicochemical characteristics, in addition to dose control and lower adverse effects [37,38,39]. Lipid nanocarriers, such as solid lipid nanoparticles (SLN), nanostructured lipid carriers (NLC), and nanoemulsions (NE), are well-known by researchers. Since their creation in the nineties, the number of research groups studying these systems has been growing. Reasons for this are their easily accessible production methods and advantages over other colloidal carriers, particularly those of low toxicity. These dispersions are heterogeneous systems with an inner lipid phase and an external aqueous phase, stabilized with one or two surfactants. Although all are composed of lipids, they differ in terms of the physical state of the lipid and the composition of the molecule [40,41,42]. Unlike nanoemulsions, lipid nanoparticles have an inner solid lipid phase since these nanoparticles are totally (for SLN) or mainly (for NLC) composed of lipids that are solid at room temperature. The wide variety of lipids used in topical lipid nanoformulations may be classified as fatty acids, waxes, steroids, partial glycerides, and triglycerides (i.e., Medium Chain Triglycerides (MCT), olive oil, fish oil, soybean oil, stearic acid, glyceryl monostearate (MEG), shea butter, candelilla wax among others) and have been granted GRAS (Generally Recognized As Safe) status by regulatory bodies [43,44]. Thus, some characteristics related to the drug incorporation rate, controlled release, and permeation into the skin could vary between different types of nanocarriers. NE have the advantage of greater drug incorporation and faster release; SLN have a controlled drug release but a lower encapsulation rate, while NLC allow greater drug incorporation than solid lipid nanoparticles, but less than nanoemulsions, and can have some controlled drug delivery [37,42,45,46,47,48].

Previous studies by the research group carried out tests with different proportions of solid and liquid lipids for the production of SLN, NLC, and NE using high-pressure homogenization and hot solvent diffusion techniques. In these studies, it was possible to identify that the best ratio for the preparation of NCL would be 70:30 (solid:liquid lipid) and that NCL and NE are the nanocarriers that show the best results in different methodologies [49,50,51].

The present work aimed to develop and compare two types of lipid nanocarriers—NE and NLC-loaded curcumin—to verify, according to several methodologies, which of the two nanocarriers would be the best for application as a promising platform for skin burn treatment.

## 2. Materials and Methods

### 2.1. Raw Materials

Curcumin, medium chain triglycerides (MCT), span^®^ 80, and tween^®^ 80 were purchased from Sigma-Aldrich (St. Louis, MO, USA). Glyceryl monostearate (MEG) and stearic acid were purchased from Alpha Química (Rio Grande do Sul, Brazil). Shea butter and candelilla wax were purchased from GM ceras (São Paulo, Brazil) and are classified by the Food and Drug Administration (FDA) as GRAS compounds. Candelilla wax is a hard and breakable wax extracted from the wax cover of the stalks of candelillas shrubs (generally of *Euphorbia cerifera* or *Euphorbia antisyphilitica*). It consists of hydrocarbons (approximately 50%, from C29 to C33), free fatty acids of alcohols and resins, but a relatively low quantity of volatile esters. Shea butter is a natural product obtained from the *Vitellaria paradoxa* tree. The main component of Shea butter is triglycerides that have oleic, linoleic, stearic, and palmitic fatty acids in addition to some unsaponifed matter such as tocopherol, sterols, and phenols. HPLC-grade acetonitrile was purchased from Pareac^®^ (Barcelona, Spain), and HPLC-grade water was prepared using a Milli-Q system by Millipore^®^ (Burlington, MA, USA). Polyethylene glycol 400 was purchased from Synth (São Paulo, Brazil), formaldehyde from Neon (São Paulo, Brazil), and purified paraffin from Procito (Porto Alegre, Brazil). Fibroblasts were purchased from the Rio de Janeiro Cell Bank, the MTT from Sigma-Aldrich (St. Louis, MO, USA), and DMSO from Synth (São Paulo, Brazil). DMEM high glucose culture medium, fetal bovine serum, and trypsin were purchased from Gibco (São Paulo, Brazil). The *P. aeruginosa* strain used was ATCC 15442, Muller Hinton broth from Himedia, and Gentamicin from Sigma-Aldrich. Ethanol, acetonitrile, phosphoric acid, and all other reagents used were of analytical grade.

### 2.2. Methods

#### 2.2.1. Selection of Lipids Using Differential Scanning Calorimetry (DSC)

The thermal properties of mixtures of solid and liquid lipids using DSC were evaluated before the production of NLC to choose the solid lipid to be used in the formulation. For the selection of lipids, we take into account their use for topical application and their toxicity. Stearic acid, glyceryl monostearate (MEG), shea butter, and candelilla wax, combined with medium chain triglycerides (MCT), were tested to study the interaction between lipids in a 70:30 ratio of solid and liquid lipids. This ratio was chosen on the basis of previous studies [49,50,51]. The lipids were heated to 80 °C and cooled to solidify again to prepare the physical mixture. After solidification, 2 mg of the lipid mixture was added to a closed aluminum pan. The experiments were performed in a DSC-60 (Shimadzu, Kyoto, Japan), with a temperature range of 20–100 °C, heated at a rate of 10 °C/min. The TA-60 WS software version 1.5 by Shimadzu Corporation was used for the analysis of thermograms, and the percentage of melting point decrease of the physical mixture was calculated. The cut-off point used as a criterion for the inclusion of the physical mixture of lipids in the production of NLC was 57 °C since the equipment used for the production of nanocarriers, the high-pressure homogenizer, has the limitation of not exceeding 60 °C. Therefore, any lipid with a melting point above 57 °C would render production unfeasible using this technique.

#### 2.2.2. Preparation of Nanoemulsions and Nanostructured Lipid Carriers

The nanoemulsions (NE) and nanostructured lipid carriers (NLC) were prepared using the high-pressure homogenization (HAP) technique [52]. For the preparation, as shown in Table 1, the oil phase was composed of medium chain triglycerides (MCT) for NE, and MCT and glyceryl monostearate (MEG) for NLC and Span 80, and heated to 60 °C. For the samples containing curcumin, this compound was also added to the oil phase. The aqueous phase containing ultrapure water and Tween 80 was also heated to 60 °C. After the complete solubilization of both phases, the aqueous phase was added to the oil phase under constant magnetic stirring (1100 rpm). The suspension formed was pre-homogenized using an Ultra-Turrax^®^ T10 basic (IKA, Baden-Württemberg, Germany) at 14,500 rpm for 2 min. Then, the resulting formulation was subjected to 6 cycles (20 s each) at 10.000 psi using an EmulsiFlex-C3 homogenizer (Avestin, Ottawa, ON, Canada).

#### 2.2.3. Particle Size and Zeta Potential

Particle size and polydispersity index of the formulations were determined by dynamic light scattering using a Zetasizer 45 Nano Series ZS90 (Malvern Instruments, Worcestershire, UK) [50,53]. Particle size was performed at a detection angle of 90°. The Stokes-Einstein equation was used to determine the hydrodynamic radius. For both analyses, samples were diluted in ultrapure water for the procedure. Zeta potential was determined by electrophoretic light scattering using a Zetasizer Nano Series (Malvern Instruments, Worcestershire, UK) [50,53]. The samples were placed in an electrophoretic cell, and an alternating voltage of ±150 mV was applied. The analyses were performed in triplicate at 25 °C. The pH assessment was performed directly using a pH meter (Model HI5221, Hannah, Kemijärvi, Finland) at room temperature, and the samples were analyzed in triplicate. The results were expressed as the average of three independent determinations.

### 2.3. Stability Assay

To monitor the stability of lipid nanocarriers, an accelerated stability study was carried out [54]. For this, the nanocarriers remained at three temperatures (4 °C, 25 °C, and 37 °C) as a function of time (zero, 15, 30, 60, 90, and 120 days), where physicochemical characteristics such as the size of the particles, zeta potential, polydispersion index, and pH as described above. In addition, centrifugation was performed for 30 min at 15,000 rpm to evaluate the possible phase separation [54].

### 2.4. Determination of Curcumin Content and Encapsulation Efficiency

The CUR content was performed in a Synergy Mx Multi-Mode reader spectrophotometer (BioTek, Winooski, VT, USA), using a quartz plate and detection at 425 nm. The CUR content (total concentration) in the nanocarrier was calculated after determining the drug concentration in the methanolic solutions and was expressed in μg of CUR/mL of nanocarrier. The CUR recovery was calculated as the percentage of the total drug concentration found in the nanocarrier in relation to the initial added amount. Encapsulation efficiency was evaluated by passing the formulations through bio-rad columns (10 kDa). After separation, the free drug was quantified by measuring absorbance in a spectrophotometer with a wavelength of 425 nm [53,55]. The encapsulation efficiency (%) was estimated as the difference between the total concentration of CUR before and after passage on Bio-rad columns. All samples were analyzed in triplicate.

### 2.5. In Vitro Release Assay 

The in vitro release study of curcumin from NE-CUR and NLC-CUR was performed using the dialysis method. For the experiments, 2 mL of each formulation were added to Sigma-Aldrich^®^ MWCO 10.000 Da dialysis bags (St. Louis, MO, USA) and placed in a beaker containing 200 mL of acidic water release medium: PEG 400 (70:30, *v*/*v*) to maintain sink conditions. The release medium was kept at 37 °C under magnetic stirring at 70 rpm. Aliquots of the release medium were collected at time intervals of 0.5, 1, 2, 4, 6, 8, 24, 48, and 72 h. The release medium was immediately replenished after each collection. The amount of curcumin in the samples was determined using the HPLC Perkin Elmer Flexar HPLC System (Perkin Elmer Inc., Shelton, CT, USA), equipped with a quaternary pump, photodiode array detector, and automatic injection with a 15 µL loop. The detector was set at 425 nm, and a computer automatically integrated peak areas. The experiments were conducted using a reversed-phase Zorbax ODS (Agilent Technologies, Wilmington, DE, USA) C18 column (150 mm × 4.6 mm I.D., with a particle size of 5 μm), maintained at 40 ± 1 °C. The mobile phase consisted of a 1% phosphoric acid: acetonitrile mixture (45:55 *v*/*v*; pH 2.7) and was eluted isocratically at a flow rate of 1 mL/min) [53,55].

The experiment was performed in triplicate for each formulation evaluated and took place in a dark environment to protect the sample from light [53]. The amounts of curcumin released were expressed in % and plotted against time (h). The data were fitted to the zero-order, first-order, and Higuchi models according to the below formulas in order to evaluate the release kinetics:

Q = Q_0_ + Kt   (Zero-order model) (1)

lnQ = lnQ_0_ − Kt   (First-order model) (2)

(3) $$\;Q=\frac{K1}{2t}\;\;\;(\mathrm{Higuchi}\;\mathrm{model})$$

In the above, Q is the amount of drug released in time t, Q_0_ is the initial concentration of the drug, and K is the model release constant.

The residual sum of squares was also presented. This parameter allows us to check whether the proposed mathematical model is well-fitted to the data. When the same scale is used, it can be interpreted that the smallest residual sum of squares indicates the mathematical model in which the data are best fitted in a linear regression.

### 2.6. Ex Vivo Skin Permeation and Retention Studies

The permeation/retention studies for the formulations were carried out using Franz diffusion cells. Porcine ear skin was used as a membrane. The porcine ears were obtained from a slaughterhouse located in the city of Pelotas/RS. The subcutaneous tissue and ear hairs were removed with the aid of scissors and a scalpel, the ears were cut into circular pieces, and skin samples were stored at −20 °C for no longer than one month. On the day of the experiment, the skin samples were left in contact with phosphate buffer (pH 7.4) for 30 min. The porcine ear skins used in the skin permeation/retention study were divided into two groups—intact skin and scalded skin—which was left in hot water at 65 °C for 8 min mimicking a first-degree burn by hot water (condition of skin layers monitored with histology). Skin pieces were placed on the Franz cells, maintaining contact with the acceptor fluid as 350 μL of formulation was added to them. The bath temperature was set at 37 °C, and the acceptor fluid used was pH 6.4 phosphate buffer with 30% PEG 400, remaining under constant stirring at 450 rpm [37]. Samples were collected from the acceptor media at 30 min, 1 h, 2 h, 4 h, 6 h, and 8 h, being replaced with the same volume of fresh media. Sink conditions were maintained during the 8 h of the experiment. At the end, the excess formulation was removed from the skin, and the skin layers were separated using a scalpel (viable epidermis and dermis), cut into small pieces, and placed in individual tubes. In order to extract the CUR from the skin, 1 mL of acetonitrile was added to each tube, which was then taken to an ultrasonic bath for 30 min. Samples were then filtered (0.22 μm membrane) and analyzed using the HPLC method previously described in Section 2.5. Results were expressed as the mean of six replicates.

### 2.7. Confocal Laser Scanning Microscopy

Confocal laser scanning microscopy was used to visualize CUR in the skin after an ex vivo permeation/retention study, an adapted method by Vaz et al., 2017 [53]. Another skin sample was also placed in the Franz diffusion cell for 8 h, and at the end of the experiment, the entire skin was removed and fixed with 4% paraformaldehyde for 6 h, then kept in a buffer phosphate with a pH of 7.4. Transverse sections of 25 microns were performed in the tissues embedded in freezing gel (Jung–Tissue Freezing Medium^®^) using a cryostat (Leica, St. Gallen, Switzerland) at a temperature of −27 °C, at CEME-Sul/FURG. The sections were placed on glass slides and kept in 70% ethanol and 0.25% ammonia solution for 1 h, followed by 10 min in 50% ethanol. Subsequently, the slides were washed with PBS pH 7.4, and a glass coverslip was placed over the sample for further analysis using immersion oil. The samples were then washed with a phosphate buffer with a pH of 7.4. Skin thickness was optically scanned at approximately 10 um increments through the *Z*-axis of a Leica confocal microscope. Optical excitation was conducted with a 488 nm argon laser beam, and fluorescence emission was detected at 500 to 550 nm.

### 2.8. Cell Viability Study

The cytotoxicity of the nanocarriers was evaluated in human skin fibroblasts (HFF-1 line) using the MTT method, which measures mitochondrial dehydrogenase activity by reducing (3-(4,5-dimethylthiazol-2yl)-2,5-diphenyl tetrazolium bromide) to formazan [56,57,58]. The HFF-1 cell line was maintained according to ATCC recommendations: dulbecco’s modified eagle’s medium (DMEM), 10% (*v*/*v*) of fetal bovine serum (FBS), 1% (*v*/*v*) of penicillin/streptomycin solution and 1%(*v*/*v*) of amphotericin B solution. The cells were maintained at 37 °C in a humidified atmosphere of 5% CO_2_ in the air. The culture medium was refreshed every 2–3 days. For cell viability assay, cells were seeded at a density of 1 × 10^5^ cells/well on 96-well tissue culture polystyrene plates and incubated overnight to promote cell adhesion. The cells were exposed to different concentrations of NE-B, NE-CUR, and NLC-CUR and incubated for 24 h. MTT solution (0.5 mg/mL) was then added to cells and incubated for 2 h at 37 °C. After this period, 150 μL of DMSO was added to dissolve the formazan crystals. Absorbance was determined at 490 nm in a Perkin Elmer microplate reader. The results are expressed as a percentage of the control (non-treated group), where the cell viability was determined by calculating according to the equation:

(4) $$cell\;viability\;\%=\frac{average\;absorbance}{average\;absorbance\;of\;the\;control}\times 100$$

### 2.9. Antimicrobial Activity Assays

For the in vitro assays to evaluate the antimicrobial activity, the excipients, free curcumin, and the NE and NLC nanocarriers with and without curcumin were tested against *P. aeruginosa* (ATCC 15442). The sterility controls of the culture medium (Muller Hinton broth—Himedia^®^*,* Kennett Square, PA, USA), the sensitivity of the ATCC strain (using antibiotic Gentamicin—Sigma^®^), the sterility of the compounds, and the positive viability control of the test microorganism were also evaluated.

The methodology recommended by the Clinical And Laboratory Standards Institute [59] was used to determine the minimum inhibitory concentration (MIC). A serial microdilution (1:2) of the compounds was carried out in Muller Hinton broth so that the concentrations varied according to the amount of curcumin in the NE and NLC (μg/mL). The plate was incubated for 24 h at a temperature of 36 °C ± 1, and after this period, resazurin (0.02%), an indicator of cell viability, was added [60,61]. The plate was again incubated at 36 °C ± 1 for 1.5 h for further reading in the spectrophotometer at a wavelength of 600 nm.

The in vitro association of curcumin, NE, and NLC with the antibiotic gentamicin was performed using the checkerboard technique, as described by Bellio et al., 2021 [62], with some modifications. The interpretation of the checkerboard results was performed through the fractional inhibitory concentration index (FICI) obtained using the following equation:

(5) $$\mathrm{FICI}=\left(\frac{MIC\;of\;compound\;A\;combined}{MIC\;of\;compound\;A\;alone}\right)+\left(\frac{MIC\;of\;compound\;B\;combined}{MIC\;of\;compound\;B\;alone}\right)$$

Fractional Inhibitory Concentration (FIC) was defined as the lowest concentration at which the two compounds in an association can inhibit bacterial growth. The FICI results were interpreted so that: FICI < 0.5 = synergism, 0.5 < FICI ≤ 1 = additivity, 1 < FICI ≤ 2 = indifference, and FICI > 2 = antagonism.

From the minimum inhibitory concentration, bacterial growth kinetics was performed in the presence of the formulations, and colonies were counted and expressed in the Colony Forming Unit (CFU). The percentage of growth was calculated according to the positive control at times 0, 1, 3, 4, 6, 24, and 48 h.

### 2.10. Statistical Analysis

Statistical analysis was performed using analysis of variance (ANOVA); a *p*-value of less than 0.05 (*p* < 0.05) was considered statistically significant. All statistical analyses were performed using Prism software (ver. 8.4.3, Graph-Pad Inc., San Diego, CA, USA).

## 3. Results

### 3.1. Compounds Selection and Stability Test

The compound selection in pharmaceutical forms is very important since they provide physical and chemical stability and improve the biopharmaceutical characteristics of drugs [63]. The evaluation of thermal properties was conducted using DSC to select the lipids on the basis of whether the liquid lipids would have the ability to disrupt the crystalline structure of the solid lipids. The melting points of the pure compounds and their physical mixtures at a ratio of 70:30 (solid lipid: liquid lipid) can be seen in Table 2. The addition of the medium-chain triglycerides (MCT) to the solid lipids (Candelilla wax, Stearic Acid, and MEG) decreased their melting point, indicating a change in the crystalline structure of the lipid. Among the solid lipids tested, MEG was the compound that showed the highest decrease in the melting point (6.82%), and it was under 57 °C. Therefore, in the following studies, MEG was used as the solid lipid, and MCT was used as the liquid lipid.

For the selection of the surfactants, tween 80 and span 80, non-ionic surfactants, were used since they are generally less irritating and more tolerated by the skin than anionic or cationic surfactants, being a safe class as surfactants to aid in the solubilization of lipophilic active compounds. Also, they are low-cost and have been used in therapeutic products for topical use to increase the permeation flux of drugs in the skin. When they are used in combination, it is possible to produce nanocarriers with lower surfactant concentrations. Stable nanocarriers are best formulated with emulsifiers or a combination of emulsifiers having HLB (Hydrophile-Lipophile Balance) values close to that required of the oil phase [64,65,66,67].

Regarding the stability of the formulations, a small increase in size was observed in the NE at all temperatures, but the PDI did not change (Table 3). On the other hand, for NCL, a greater difference in sizes over time can be observed, but in the same way, the PDI did not vary (Table 4). The zeta potential of both formulations showed no substantial changes. For pH, a slight decrease was observed in NE, and no difference was observed in NCL. Regarding the encapsulation efficiency, it can be observed that NE have a higher initial encapsulation efficiency than NCLs and that in both cases, a slight decrease in this value was observed after 120 days at all temperatures tested. In addition, the formulations were submitted to centrifugation at all times of the experiment, and there was no phase separation in any of the periods.

### 3.2. In Vitro Release Assay

**Figure 1:**
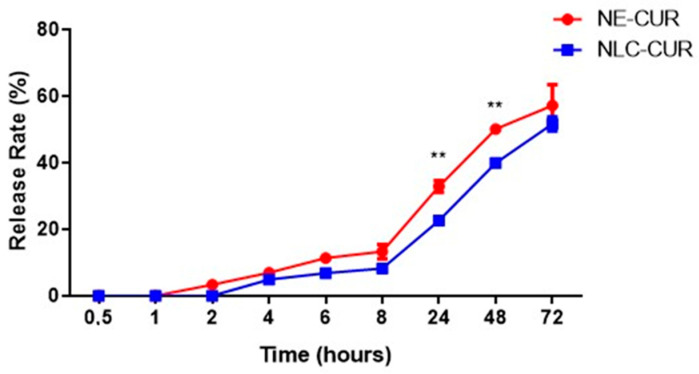
Cumulative percentage of curcumin released from NE-CUR and NLC-CUR. Statistical analysis was performed using ANOVA. ** *p* ≤ 0.005.

Figure 1 shows the in vitro release profile of curcumin from the formulations in distilled water: PEG 400 (70:30, *v*/*v*, pH 4.0) at 37 °C. Release experiments were carried out at pH 4.0 due to the low stability of curcumin in neutral and basic pH values. The experiments were carried out in sink conditions since the maximum concentration of CUR reached in the release medium corresponded to 10% of its saturation concentration (the solubility of curcumin in the release medium was 0.099 mg/mL). When the NE-CUR formulation was evaluated, measurable amounts of curcumin were detected after 2 h. For NLC-CUR, it was possible to quantify curcumin after 4 h. In Figure 1, it can be seen that there was a statistical difference between the amounts of curcumin released by the formulations at 24 h and 48 h. In 72 h, NE-CUR released 57.14% of curcumin, and NLC-CUR released 51.62%. In the analysis of the CUR release kinetics from formulations, all times were used to construct the graphs and perform the linear regression since, in the 24 h period, the release plateau was not observed.

**Figure 2:**
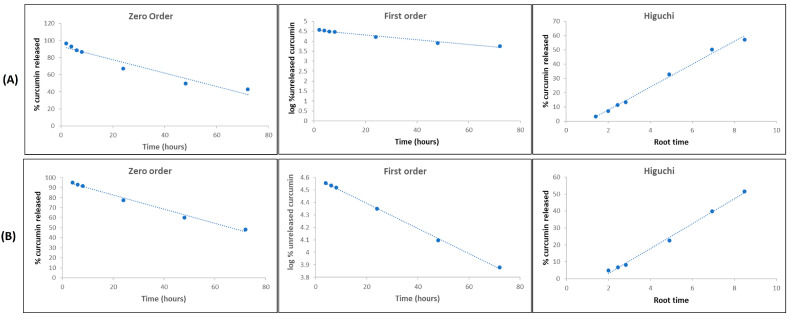
Release kinetics from (**A**) NE-CUR and (**B**) NLC-CUR.

Data from the in vitro release study were fitted to three different mathematical models: zero order, first-order, and Higuchi [68,69] (Figure 2). The evaluation showed that for NE-CUR, the data fit better in the Higuchi model (r^2^ = 0.99), demonstrating that the release is controlled by the drug diffusion process through the nanocarrier matrix (Table 5). For NLC-CUR, it was not possible to differentiate between the first-order and Higuchi models. The release constant (K) values of NE-CUR and NLC-CUR, considering the Higuchi model, were 0.04 and 0.036, respectively (Table 5).

### 3.3. Ex Vivo Skin Permeation and Retention Studies

Table 6 shows the results of permeation/retention of curcumin on intact or scalded skin to simulate a burn. When we analyzed the epidermis of intact skin, 0.94 μg/cm^2^ of curcumin (NE-CUR) and 1.17 μg/cm^2^ of curcumin (NLC-CUR) were retained, while in the epidermis of the scalded skin, 4.21 μg/cm^2^ of curcumin (NE-CUR) and 5.08 μg/cm^2^ (NLC-CUR) were retained. Regarding the dermis of intact skin, the amount of curcumin retained was 1.20 μg/cm^2^ for NE-CUR and 0.87 μg/cm^2^ for NLC-CUR, and in the dermis of scalded skin, the amount of curcumin retained was 4.97 μg/cm^2^ for NE-CUR and 3.55 μg/cm^2^ for NLC-CUR. These data indicate that NE-CUR has better penetration into the skin (Intact or scalded). We did not detect curcumin in the acceptor fluid for either intact or scalded skin, indicating the preferential accumulation of curcumin in the skin layers and that the nanocarriers did not cross the skin.

### 3.4. Confocal Laser Scanning Microscopy

**Figure 3:**
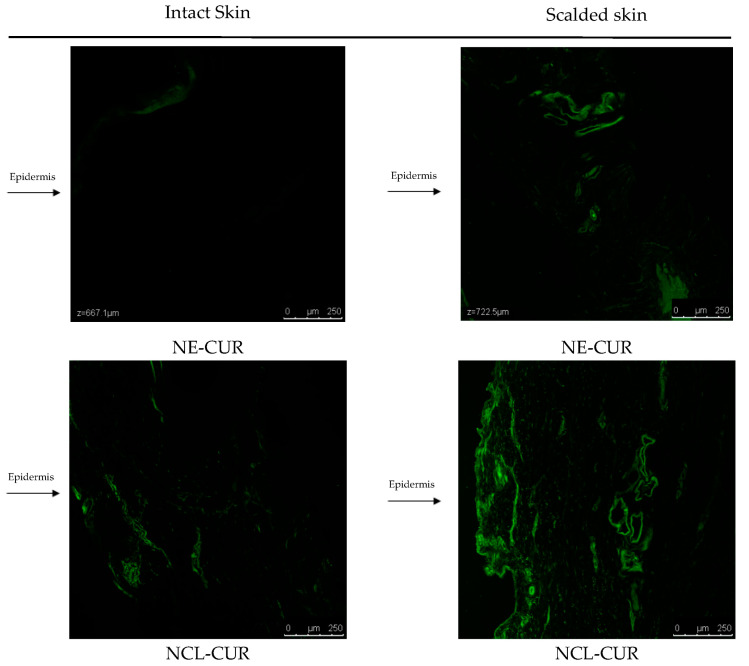
Confocal microscopy images of NE-CUR and NCL-CUR in intact and scalded pig ear skins.

Confocal laser scanning microscopies are presented in Figure 3. It could be observed that there was higher retention of both formulations in scalded skin and that NLC-CUR remains in higher amounts in the epidermis when compared to NE-CUR, corroborating the data presented in the Franz cell retention assay.

### 3.5. Cytotoxicity Assay

**Figure 4:**
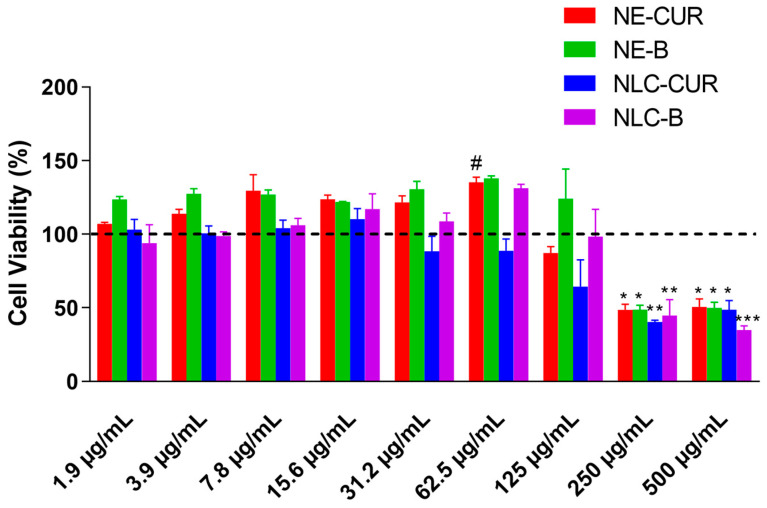
Effects of NE-CUR, NE-B, NLC-CUR, and NLC-B on cellular viability of HFF-1 cells. Each bar represents the mean +/− SEM (standard error of mean) of percentage of cell viability. Control (untreated cells) is represented by the dotted line. The experiments were performed in duplicate. Statistical analysis was performed using one-way ANOVA followed by Bonferroni’s multiple comparisons test. * *p* < 0.05; ** *p* < 0.01; *** *p* < 0.001 represent the difference from control (dotted line). # *p* < 0.05 represent the difference from NLC-CUR.

The results obtained through the MTT assay (Figure 4) showed cell viability after treatment with NE-CUR and NLC-CUR up to 125 μg/mL. Above these concentrations, all the formulations were toxic to the cells. It was also observed that at a concentration of 62.5 μg/mL, there was a statistical difference between the formulations NE-CUR and NLC-CUR with the same curcumin concentration. This indicates that there are more viable cells when the cells are treated with NE-CUR than with NLC-CUR at this concentration.

### 3.6. Antimicrobial Activity Assays

In antimicrobial activity studies, the compounds were tested at the concentrations used for the production of nanocarriers: liquid and solid lipids, MCT (70 mg/mL) and MEG (49 mg/mL), as well as the aqueous and oil phase surfactants, Tween 80 (20 mg/mL) and Span 80 (30 mg/mL), and none showed antimicrobial activity in the microdilution assay after 24 h of incubation.

Free curcumin showed antimicrobial activity against the *P. aeruginosa* strain with a MIC of 57.7 µg/mL, while gentamicin (used as a control in the tests) showed a MIC of 0.125 µg/mL. All treatments showed a statistical difference after 48 h of exposure compared to the untreated control (*p* < 0.05). Furthermore, in addition to gentamicin, all compounds evaluated (NLC-CUR, NE-CUR, or CUR) inhibited *P. aeruginosa,* and no significant differences were observed between treatments (*p* > 0.05).

**Figure 5:**
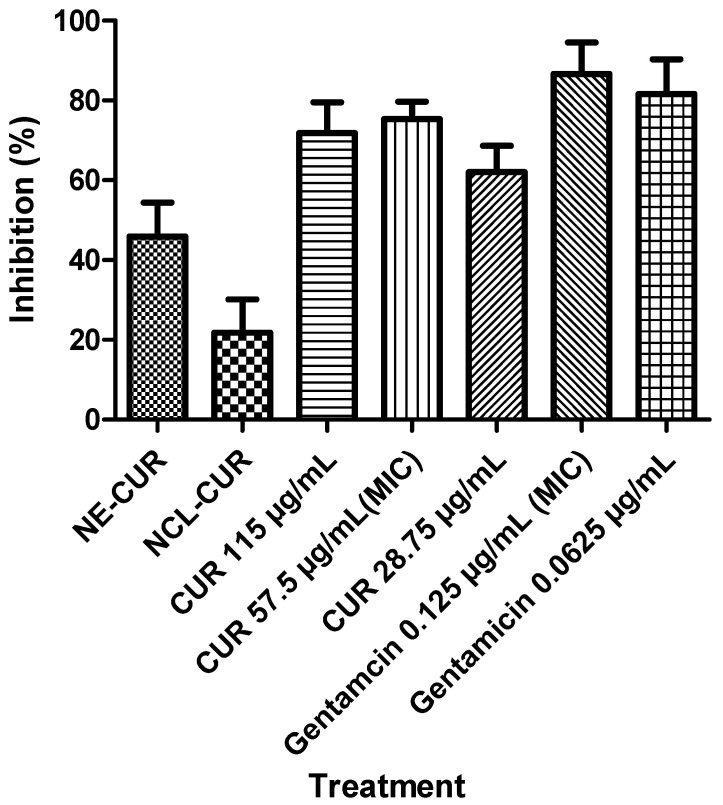
Percentage of bacterial inhibition. NE-CUR = Nanoemulsion containing 460 µg/mL of curcumin, NCL-CUR = Nanostructured lipid carrier containing 460 µg/mL of curcumin. All data are shown as the mean ± S.E.M, and all treatments showed statistical differences in relation to the untreated control (*p* < 0.05). We did not have a significant difference between treatments with different concentrations.

The strain was exposed to the compounds for up to 48 h to evaluate the bacterial growth kinetics of *P. aeruginosa*. The colonies expressed in CFU were counted (Figure 5), and the percentage of growth was calculated according to the positive control. Gentamicin (0.125 µg/mL/MIC value) was used as a control and completely inhibited bacterial growth after 3 h, while free curcumin (57.5 µg/mL/MIC value) reduced bacterial growth by 75% in 3 h. On the other hand, nanocarriers seem to have different behavior since NE-CUR inhibited bacterial growth by 65% after 24 h, while NLC-CUR showed lower inhibition at all times when compared to NE-CUR.

**Figure 6:**
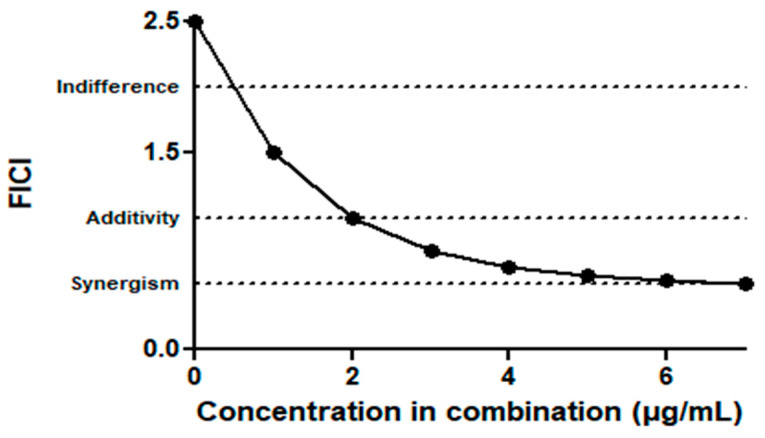
Curve of the lowest concentrations of Curcumin with Gentamicin (0.06 µg/mL) in combination, capable of inhibiting bacterial growth, expressed using the fractional inhibitory concentration index (FICI). Concentrations from 115 to 0.9 µg/mL (represented by 0 to 7 on the *x*-axis).

Regarding the test with the association between the compounds, it was observed that concentrations of curcumin showed an additive and synergistic effect when associated with gentamicin. In contrast, when NE-CUR was associated with gentamicin, the interaction was indifferent, but this association halved the MIC of the antibiotic gentamicin (Table 7). Interestingly, there was a positive interaction in the association of the subinhibitory concentration of gentamicin (0.06 µg/mL) with all evaluated concentrations of curcumin. Furthermore, the concentration necessary for curcumin to act synergistically with the antimicrobial is 12 times lower than that identified as MIC (0.9 µg/mL) (Figure 6).

## 4. Discussion

Different types of materials, like polymeric, lipid, and inorganic materials, have been used to produce nanomaterials. Among these, lipid nanocarriers have considerable advantages due to their biocompatibility, biodegradability, low toxicity, and scale-up capacity. Intralipid^®^ was the first safe lipid parental emulsion developed in the 1960s. In the same decade, liposomes have become the traditional models for lipid-based formulation. However, the limited physical stability of the liposomal suspension, drug leakage, low targeting ability, non-specific clearance by monocytes and macrophages, and up-scaling difficulties are disadvantages of this system. Several other carrier systems of a lipidic nature were developed, such as nanoemulsions (NE), solid lipid nanoparticles (SLN), and nanostructured lipid carriers (NCL) [70,71]. SLN were produced when solid lipids at room temperature were used; when liquid lipids were utilized, NE were formed; and when a mixture of liquid and solid lipids was used, NLC was produced [72,73,74,75]. Thus, although they are all composed of lipids, they differ in terms of the physical state of the lipid and the composition of the molecule [40]. These differences can greatly impact the physicochemical properties of the nanocarrier formulation, such as particle size, drug loading, release rate, and skin permeation capability [76].

In this study, we develop and compare two types of lipids nanocarriers, NE and NLC, with curcumin as a model of a lipophilic drug, to verify, according to several methodologies, which of the two nanocarriers would be the best for application as a promising platform for skin burn treatment. Although other types of lipid nanocarriers containing curcumin have already been developed [53,77,78,79], no study makes a careful selection of excipients, compares the two types of nanomaterials most used for dermatological application (NE and NCL), and performs permeation/retention tests on intact skin that mimicks a 1-degree burn to be used as a platform for treating skin burns; thus, demonstrating the novelty of this work.

During the development of nanocarriers, solid and liquid lipid matrices assist in the solubilization or the dispersion of the drug. In the case of NCL, an understanding of the interaction between lipids with different physical states after heating and cooling is fundamental for the manufacture of nanocarriers. A high-pressure homogenizer (HPH), which is used for the preparation of nanocarriers, has the limitation of not exceeding the temperature of 60 °C; therefore, any lipid with a melting point above 57 °C is not compatible with this technique [51]. In Table 1, it is possible to observe the percentage of melting point decrease after DSC analysis. The results suggest that mixtures of MEG with MCT are suitable for producing NLC using HPH. The decrease in melting point for this physical mixture (6.82%) may indicate an interaction between these compounds and, therefore, a disorder in the lipid matrix. The entrapment of a liquid lipid into the solid form of the nanocarriers may increase the number of imperfections in the core of this matrix and facilitate the encapsulation of a more substantial amount of drug while maintaining prolonged release from the nanocarrier [51,80].

After the initial choice of the lipids, two types of nanocarriers were produced: NE and NLC. The average size results of the formulations, around 190 nm and 219 nm for NE-CUR and NLC-CUR, respectively, were considered satisfactory for topical formulations. The polydispersity index of less than 0.3 indicates that the nanocarriers have a narrow particle size distribution [81,82], and the zeta potential was also satisfactory, indicating electrostatic stability. For NE-CUR, the values corroborated with a study carried out of curcumin-loading nanoemulsions, with a size of 195–217 nm [83], and with another study where nanoemulsions were produced for the treatment of wounds, with an average size between 150 and 230 nm [84]. Regarding NLC-CUR, the sizes found in the literature are larger than those found in the present study. A study by Lee et al. (2020) [85] produced NLC containing curcumin and epidermal growth factor with an average size of 331.8 nm and PDI of 0.31 for the treatment of chronic wounds. The study performed by Vijayakumar et al. (2019) [86] produced NLC modified with ginsenoside containing curcumin using the melt emulsification technique and obtained particles of 340 nm and PDI of 0.17. The encapsulation efficiency was around 95% for NE-CUR and 92% for NLC-CUR. Although the difference is small, it is well described in the literature that the encapsulation efficiency can be influenced by the composition of the nanoparticles, as observed by Keck et al. (2021) [73], where mixed lipid matrices, composed of solid and liquid lipids, showed a decrease in curcumin content when compared to formulations composed of a liquid lipid.

Stability is one of the critical aspects in ensuring the safety and efficacy of nanomaterials. The stability issues of drug nanoparticles could arise during manufacturing, storage, and shipping. For instance, the high pressure or temperature produced during manufacturing can cause crystallinity changes to the drug particles. Storage and shipping of the drug products may also bring about a variety of stability problems, such as sedimentation, agglomeration, and crystal growth. Therefore, stability issues deserve significant attention during pharmaceutical product development [87,88,89,90]. In the present study, the nanocarriers were evaluated for more than 120 days at different temperatures (4 °C, 25 °C, and 37 °C). The formulations proved to be stable during this period, and also, after centrifugation, no phase separation was tested at all times. Only small changes in size and encapsulation efficiency were observed, mainly for NLC at a temperature of 37 °C. A study carried out with curcumin lipid nanoemulsions demonstrated that they were stable for 60 days at a temperature of 4 °C [91]. The study developed by Azami et al., 2018 [92] evaluated the stability of NE-CUR by monitoring the change in phase separation, creaming, and discoloration after storage for 2 months at room temperature, and the determination of particle size and zeta potential was investigated after three cycles freezing and thawing. As a result, it was observed that no significant changes were found in particle size and zeta potential, even after three cycles of freezing and thawing. Another study performed with nanoparticles containing curcumin observed stability for three months at room temperature, keeping the sizes unchanged in all formulations, NE, NLC, and NLS [73].

An in vitro release assay was performed to understand the curcumin release from the NE-CUR and NLC-CUR formulations. With the data obtained, the release profiles were traced, as well as the mathematical model that best explains the release, considering the zero-order, first-order, and Higuchi models [93,94,95]. NE-CUR showed a behavior based on the Higuchi model, and NLC-CUR exhibited a release profile that fitted the first-order and Higuchi models. The first-order model adjusts to release profiles that are influenced by the amount of drug in the formulation, that is, the concentration gradient. The Higuchi model, in turn, describes profiles whose release is mainly related to drug diffusion from formulations, being one of the main mathematical models applied for controlled release [96,97]. In this sense, when release profiles fit the Higuchi model, as in this work, it is suggested that the formulation exerts some control over drug release. The magnitude of this control can be inferred using the Higuchi release constant (K). For the NE-CUR and NLC-CUR formulations, k values of 0.040 and 0.036 were obtained, respectively, indicating greater control of the release by the NLC-CUR formulation. Although the difference between the Higuchi constants was subtle, the direct analysis of the release profiles clearly shows the difference between the NE-CUR and NLC-CUR formulations. In addition to the slower release of the NLC-CUR formulation at the beginning of the trial, reflecting the quantification of curcumin only 4 h after the beginning of the evaluations, a statistical difference (*p* < 0.005) was obtained between the two formulations at the times of 24 and 48 h. Lower curcumin concentration was also observed in the release medium at 72 h for the NLC-CUR formulation. As both formulations showed the same encapsulation efficiency and, therefore, presented the same initial concentration of curcumin, the differences in the release profiles through the concentration gradient cannot be justified.

It is safe to affirm that the different release behaviors are due to the structural differences between NE-CUR and NLC-CUR, with greater control of release by NLC-CUR. Such results can be compared with the in vitro release study carried out by Liakopoulou et al. (2020) [98], where lipid nanocarriers with curcumin showed a rapid release of curcumin within 4 h, followed by a sustained release within 32 h. The NE showed higher release when compared to SLN and NLC, where NE, NLC, and SLN had released 66.6%, 44.79%, and 35.19% of CUR, respectively. This result corroborates our study and is in agreement with the literature, which states that the faster drug release from nanoemulsions is expected and may be justified by the presence of solid lipids in NLC-CUR that can slow the release of curcumin [37,46,48,73].

It is important to note that topical products must have efficiency, low toxicity, and not reach the bloodstream, so penetration and skin absorption of such products must be observed [99]. For the permeation and retention of formulations to be evaluated in vitro, the Franz diffusion cell can be used, which evaluates the skin absorption potential for a drug. This depends on the ability of nanoencapsulated materials to permeate or be retained in a certain layer of the skin. Lipid nanoparticles have the advantage of biocompatibility with the skin and reduced toxicity, which is important for the development of drugs for the treatment of burns [26,100,101]. In this study, the NE-CUR and NLC-CUR formulations did not permeate either intact or scalded skin of pig ears. This result indicates that the formulations remain in the epidermal and dermal tissues even with the skin mimicking a hot water burn, as in the case of scalded skin, which is important to avoid the systemic effect of the drug [102]. When we compare the retention results of the two nanocarriers, we can observe that NLC-CUR remains in higher concentration in the epidermis, and NE-CUR in the dermis, in intact and scalded skin. These results were likely because NLC-CUR is composed of liquid and solid lipids, finding higher resistance in penetrating deeper into the skin. However, the retention values in scalded skin are substantially higher than in intact skin, which is justified by the loss of the protective barrier composed of the stratum corneum in a burn [103]. A study carried out by Keck et al. (2021) [73] evaluated the effectiveness of dermal penetration of lipid nanoparticles with curcumin. It showed that the amount of curcumin penetrated was influenced by the increasing amount of liquid lipid in the nanocarriers when comparing SLN, NLC, and NE, demonstrating that the composition of the lipid matrix influences the dermal penetration efficacy of lipophilic drugs.

Despite promising drug delivery, there is limited knowledge about the toxicity of the nanocarriers. In general, lipid nanocarriers are composed of biocompatible and biodegradable materials; however, the interactions among nanoparticles and biological systems are influenced by nanoparticle characteristics, including size, composition, chemical functionality, and surface charge. Additionally, the types of lipids, surfactants, and solvents used to develop nanosystems are important factors that affect toxicity. In this work, we evaluate the cytotoxicity of the formulations in human fibroblast lineage (HFF-1) after 24 h of CUR exposure. The toxicity of curcumin in fibroblasts cell lines was previously described by other authors. For example, Rujirachotiwat et al. (2021) [104] determined that curcumin did not affect the cell viability of human gingival fibroblasts until 20 μM (7.3 μg/mL). Moreover, Lu et al. (2017) [105] verified that the cytotoxic and antiproliferative effects of curcumin were from 5 to 80 μM. Since we did not have information about the toxicity of our nanocarriers, we chose to perform a broad concentration-response curve starting from low concentrations of curcumin to higher concentrations. Our results showed that NE-CUR and NLC-CUR are safe at 125 μg/mL. Also, more viable cells were observed when the cells were treated with NE-CUR than with NLC-CUR at a concentration of 62.5 μg/mL. This may indicate greater cell proliferation when NE-CUR is used at this concentration. This fact may be associated with the healing property of curcumin that is related to the proliferation and migration of fibroblasts [98].

The potential of curcumin in wound healing has been explored by several researchers, and it has been suggested that its antioxidant and anti-inflammatory properties can contribute to its effect. Curcumin can accelerate wound contraction, suppress inflammatory response, enhance collagen deposition, and induce angiogenesis [28]. The re-epithelialization and the increased migration of myofibroblasts and fibroblasts were observed in a study performed with curcumin in burnt rats, demonstrating that curcumin plays an important role in the wound healing process and tissue repair in burns [29]. In addition, another study carried out on rats with burns demonstrated that curcumin was able to completely re-epithelialize wounds, with a decrease in inflammatory cells, increased fibroblast proliferation, and angiogenesis [30].

One of the most frequent nosocomial pathogens in burn injuries is *P. aeruginosa*, which is difficult to treat [33,106,107,108]. Furthermore, infections with *P. aeruginosa* are described as the main cause of morbidity and mortality among hospitalized burn patients, demonstrating the importance of studies with compounds with antimicrobial activity for the control and treatment of these infections [109,110]. Curcumin plays an important role in this case, as it has antibacterial activity against several gram-negative bacteria, such as *P. aeruginosa* [21,33,111,112]. In a study performed by Bhawana et al. (2011) [113], the authors concluded that curcumin nanoparticles act on the cell wall of bacteria, breaking it and penetrating its interior, thus disrupting the cell organelles since curcumin interacts with the external phospholipid membrane of gram-negative and gram-positive bacteria. In the present study, the antimicrobial activity of free curcumin was evaluated.

However, when evaluating the kinetics of bacterial growth of *P. aeruginosa*, 57.5 µg/mL of free curcumin reduced bacterial growth by 75% in 3 h. While NE-CUR inhibited 65% of bacterial growth in 24 h, NLC-CUR showed lower inhibition at all times. This may be due to the presence of solid lipids in their composition, which causes curcumin to be released more slowly than in nanoemulsion, where there are only liquid lipids. The result of the release of curcumin from the nanocarriers suggests that the amount released in 24 h during the MIC test (460 µg/mL) is not sufficient to inhibit bacterial growth. However, in the kinetics of growth, both NE-CUR and NLC-CUR were able to inhibit bacterial growth during the evaluated time, which may be related to the controlled and delayed release of curcumin from the nanocarriers Shariati et al. (2019) [33] performed a broth microdilution assay with free curcumin and ultrasound-prepared curcumin nanoparticles against *P. aeruginosa* and obtained 128 μg/mL of curcumin nanoparticles, while that of free curcumin was 256 μg/mL. In this same study, curcumin nanoparticles destroyed the *P. aeruginosa* biofilm, proving to be promising formulations for the treatment of burns.

We also tested the combination of curcumin and gentamicin against *P. aeruginosa*. Curcumin concentrations showed additive and synergistic effects when associated with gentamicin, whereas when it was associated with NE-CUR and gentamicin, the interaction was indifferent, but demonstrated that the association of NE-CUR and gentamicin halved the MIC of the antibiotic gentamicin. In a study by Bahari et al. (2017) [114], the MIC of curcumin against *P. aeruginosa* was 0.128 mg/mL. Using the checkerboard technique, a synergistic effect was observed between curcumin and azithromycin and between curcumin and gentamicin, corroborating with the results found in the present study.

Zheng et al. (2020) propose that the mechanism of antibacterial activity of curcumin, which inhibits bacterial growth, is the cause of oxidative stress or even acts synergistically with other antibiotic agents [115]. However, some authors have also proposed the inhibition of virulence factors, such as the inhibition of the formation of bacterial biofilms and adhesion molecules to surfaces, among others, including those against *P. aeruginosa* [116,117,118].

It is well described in the literature that small changes in the composition of formulations can alter their biopharmaceutical properties [73,119]; therefore, additional in vitro and in vivo tests are suggested for future studies to evaluate better and understand the activity of NE-CUR and NLC-CUR. However, it is worth noting that NE-CUR appear to have better biopharmaceutical characteristics for being a possible platform for the treatment of burns. Comparing the results described in this work, we can observe that NE-CUR have better stability and a better curcumin release profile, has a better potential to permeate the skin and remain in the dermis, has lower cytotoxicity, and has the potential to induce fibroblast cell proliferation, in addition to having better antimicrobial properties.

## 5. Conclusions

Formulations with nanometric size and suitable properties for dermal application were obtained, with good encapsulation efficiency, and remained stable for 120 days at different temperatures. NE-CUR demonstrated better cutaneous release and retention than NLC-CUR, showing advantages in terms of cell viability. Regarding the microbial activity assays, curcumin has activity against *P. aeruginosa,* and NE-CUR associated with gentamicin have favorable results. The results presented in this study demonstrate that NE-CUR is promising for the treatment of burns. However, they reinforce the need for further studies to verify the activity of nanoemulsions and nanostructured lipid carriers containing curcumin in the regeneration process in burn injuries.

## Figures and Tables

**Table 1:** Composition of the nanocarriers prepared using the high-pressure homogenization (HAP) technique.

Compounds (%)	Formulation			
	NE-B	NE-CUR	NLC-B	NLC-CUR
Curcumin	-	0.06	-	0.06
MCT	7.0	7.0	2.1	2.1
MEG	-	-	4.9	4.9
Span 80	3.0	3.0	3.0	3.0
Tween 80	2.0	2.0	2.0	2.0
Water q.s.p	100	100	100	100

NE-B and NLC-B: nanoemulsion and nanostructured lipid carriers without curcumin. NE-CUR and NLC-CUR: nanoemulsion and nanostructured lipid carriers with curcumin.

**Table 2:** Melting point of solid and liquid lipid mixtures was tested, and the percentage of melting point decreased.

Compounds	Melting Point (°C)	Melting Point Decrease (%)
Candelilla wax	58.91	
Candelilla wax + MCT	55.94	5.04
Stearic acid	61.7	
Stearic acid + MCT	58.8	4.7
MEG	60.23	
MEG + MCT	56.12	6.82
Shea butter	56.29	-
Shea Butter + MCT	57.03	-

**Table 3:** Stability of NE-CUR at times 0, 15, 30, 60, 90, and 120 days, at temperatures of 4 °C, 25 °C, and 37 °C.

Time (Days)	Temperature (°C)	Size (d. nm)	PDI	Zeta Potential (mV)	EE (%)	pH
0	4	191.08 ± 1.27	0.25 ± 0.02	−20.10 ± 0.49	94.53	7.45
	25	196.00 ± 2.09	0.26 ± 0.02	−19.80 ± 0.54	95.34	7.41
	37	192.05 ± 2.10	0.25 ± 0.12	−20.34 ± 0.69	94.9	7.39
15	4	192.27 ± 2.12	0.25 ± 0.03	−21.03 ± 0.65	93.2	7.40
	25	202.70 ± 1.89	0.26 ± 0.01	−21.83 ± 0.75	92.78	7.37
	37	198.35 ± 2.01	0.25 ± 0.02	−21.80 ± 0.2	92.08	7.37
30	4	199.66 ± 1.06	0.25 ± 0.02	−20.60 ± 0.22	92.44	7.14
	25	207.50 ± 1.72	0.26 ± 0.01	−19.21 ± 0.51	92.34	7.01
	37	206.75 ± 1.85	0.26 ± 0.02	−20.80 ± 0.35	92.03	7.23
60	4	198.54 ± 1.99	0.25 ± 0.01	−20.10 ± 0.29	92.32	7.12
	25	203.40 ± 2.01	0.25 ± 0.05	−21.41 ± 0.23	92.32	7.11
	37	201.43 ± 2.10	0.25 ± 0.04	−19.82 ± 0.31	91.3	7.19
90	4	199.64 ± 1.09	0.26 ± 0.02	−21.03 ± 0.13	92.01	7.09
	25	202.39 ± 1.06	0.25 ± 0.01	−21.73 ± 0.02	91.78	7.10
	37	204.51 ± 2.03	0.25 ± 0.01	−22.90 ± 0.12	91.1	6.99
120	4	200.71 ± 2.45	0.24 ± 0.02	−23.09 ± 0.22	92.07	7.01
	25	206.70 ± 2.56	0.24 ± 0.02	−20.90 ± 0.06	91.8	7.14
	37	202.03 ± 2.09	0.24 ± 0.02	−22.08 ± 0.14	91.03	6.98

**Table 4:** Stability of NLC-CUR at 0, 15, 30, 60, 90, and 120 days, at temperatures of 4 °C, 25 °C, and 37 °C.

Time (Days)	Temperature (°C)	Size (d. nm)	PDI	Zeta Potential (mV)	EE (%)	pH
0	4	212.85 ± 1.99	0.28 ± 0.04	−25.65 ± 0.93	92.45	7.00
	25	212.54 ± 1.27	0.29 ± 0.02	−26.70 ± 1.17	92.90	7.13
	37	211.09 ± 1.92	0.28 ± 0.09	−25.60 ± 0.12	92.5	7.11
15	4	210.43 ± 2.09	0.30 ± 0.02	−26.10 ± 2.01	90.66	7.12
	25	209.70 ± 2.53	0.30 ± 0.02	−26.02 ± 0.19	92.80	7.12
	37	214.90 ± 1.98	0.28 ± 0.02	−27.41 ± 2.01	92.49	7.19
30	4	220.30 ± 1.72	0.29 ± 0.01	−25.63 ± 2.08	90.12	7.09
	25	221.62 ± 1.86	0.29 ± 0.02	−25.75 ± 1.67	92.77	7.19
	37	230.08 ± 1.09	0.28 ± 0.02	−26.46 ± 1.03	91.7	7.07
60	4	229.54 ± 0.19	0.29 ± 0.03	−25.89 ± 0.21	90.1	7.00
	25	231.62 ± 1.95	0.29 ± 0.04	−28.50 ± 1.19	92.34	6.98
	37	228.90 ± 2.09	0.28 ± 0.09	−27.34 ± 0.94	91.56	7.01
90	4	231.45 ± 1.09	0.28 ± 0.07	−27.98 ± 2.02	90.04	7.05
	25	228.53 ± 1.74	0.29 ± 0.01	−28.94 ± 2.14	91.54	6.85
	37	231.45 ± 2.09	0.28 ± 0.01	−26.65 ± 1.98	90.08	6.98
120	4	230.10 ± 1.04	0.29 ± 0.09	−27.50 ± 1.84	90.05	7.01
	25	231.90 ± 2.11	0.29 ± 0.08	−28.40 ± 1.12	91.29	7.01
	37	232.43 ± 2.01	0.28 ± 0.08	−26.70 ± 0.17	90.02	6.87

**Table 5:** Kinetic parameters obtained from NE and NLC curcumin release profiles.

	Correlation Coefficient (r^2^)		Residual Sum of Squares		K (72 h) ^1^	
	NE-CUR	NLC-CUR	NE-CUR	NLC-CUR	NE-CUR	NLC-CUR
Zero order	0.94	0.98	156.54	21.66	-	-
First order	0.97	0.99	0.0152	0.002	-	-
Higuchi	0.99	0.99	20.46	8.47	0.04	0.036

^1^ The value of K was determined considering the Higuchi model for both formulations.

**Table 6:** Permeation/retention of curcumin from NE-CUR and NCL-CUR in the different skin layers of porcine ear skin using Franz-type diffusion cells.

	Intact Skin		Scalded Skin	
	NE-CUR	NCL-CUR	NE-CUR	NCL-CUR
**Epidermis (μg/cm^2^)**	0.94 ± 0.08	1.17 ± 0.61	4.21 ± 0.76 *	5.08 ± 0.24
**Dermis (μg/cm^2^)**	1.20 ± 0.46	0.87 ± 0.24	4.97 ± 1.68 **	3.55 ± 1.93
**Fluid (μg/cm^2^)**	<LOQ	<LOQ	<LOQ	<LOQ

LOQ: Limit of quantification. Analysis of variance followed by the TWO-ANOVA test (TUKEY). * NE-CUR epidermis (intact skin) vs. NE-CUR epidermis (scalded skin); ** NE-CUR dermis (intact skin) vs. NE-CUR dermis (scalded skin). * *p* < 0.05; ** *p* < 0.005.

**Table 7:** Association of curcumin and gentamicin against *P. aeruginosa* bacterial strain.

Strain	Compound	MIC Alone	Antimicrobial Activity in Combination (CUR and GEN)	FICI	Interaction *
*Pseudomonas aeruginosa*	CUR	5.5 µg/mL	28.7 µg/mL and 0.06 µg/mL	1	Additivity
			28.7 µg/mL and 0.03 µg/mL	0.75	Additivity
	GEN	0.12 µg/mL	28.7 µg/mL and 0.02 µg/mL	0.62	Additivity
			14.4 µg/mL and 0.06 µg/mL	0.75	Additivity
			7.2 µg/mL and 0.06 µg/mL	0.62	Additivity
			3.6 µg/mL and 0. 06 µg/mL	0.56	Additivity
			1.8 µg/mL and 0.06 µg/mL	0.53	Additivity
			0.9 µg/mL and 0.06 µg/mL	0.5	Synergism

* MIC values µg/mL; FICI ≤ 0.5 = synergism; 0.5 < FICI < 1 = additivity; 1 < FICI < 2 = indifference and FICI > 2 = antagonism.

## Data Availability

Data are available upon request to the corresponding author.
